# Triclabendazole Treatment Failure for *Fasciola hepatica* Infection among Preschool and School-Age Children, Cusco, Peru^1^

**DOI:** 10.3201/eid2707.203900

**Published:** 2021-07

**Authors:** Maria L. Morales, Melinda B. Tanabe, A. Clinton White, Martha Lopez, Ruben Bascope, Miguel M. Cabada

**Affiliations:** Universidad Peruana Cayetano Heredia, Cusco, Peru (M.L. Morales, A.C. White Jr., M. Lopez, M.M. Cabada);; University of Texas Medical Branch, Galveston, Texas, USA (M.B. Tanabe, A.C. White Jr., M.M. Cabada);; Peruvian Ministry of Health, Cusco (R. Bascope)

**Keywords:** triclabendazole, treatment failure, fascioliasis, Fasciola hepatica, liver fluke, trematode, parasites, infection, efficacy, drug resistance, preschool children, school-age children, antimicrobial resistance, Cusco, Peru

## Abstract

We conducted a retrospective cohort study of children who had chronic fascioliasis in the highlands of Peru to determine triclabendazole treatment efficacy. Children passing *Fasciola* eggs in stool were offered directly observed triclabendazole treatment (>1 doses of 10 mg/kg). Parasitologic cure was evaluated by using microscopy of stool 1–4 months after each treatment. A total of 146 children who had chronic fascioliasis participated in the study; 53% were female, and the mean ± SD age was 10.4 ± 3.1 years. After the first treatment, 55% of the children achieved parasitologic cure. Cure rates decreased after the second (38%), third (30%), and fourth (23%) treatments; 17 children (11.6%) did not achieve cure after 4 treatments. Higher baseline egg counts and lower socioeconomic status were associated with triclabendazole treatment failure. Decreased triclabendazole efficacy in disease-endemic communities threatens control efforts. Further research on triclabendazole resistance and new drugs to overcome it are urgently needed.

Triclabendazole is the only medication recommended by the World Health Organization for treatment of *Fasciola hepatica* liver fluke infection in humans (*1*). Triclabendazole use in human infections was initially reported in Europe in 1986 (*2*). Several studies in Bolivia, Peru, and Egypt have documented efficacy of 80%–100% after 1 or 2 doses (*3**–**5*). The recommended triclabendazole treatment regimen is 1–2 doses of 10 mg/kg with a fatty meal in patients >6 years of age (*1**,**6*). Triclabendazole has been administered at higher doses and in children <6 years of age despite limited safety and efficacy data (*3**,**8**–**10*).

The widespread use of triclabendazole in livestock that have fascioliasis has been associated with decreasing efficacy. Triclabendazole resistance in cattle was first reported from Australia in 1995 (*11*). Since then, >11 countries have reported triclabendazole resistance in livestock (*12*). A reported case of triclabendazole resistance in a farmer from the Netherlands was described in 2012 (*13*). Other human cases have been described in Chile, Peru, Portugal, and Turkey (*14**–**16*). Decreasing triclabendazole efficacy is a threat to public health and livestock industry in disease-endemic regions. However, little is known about triclabendazole treatment failure rates in human fascioliasis (*12*).

We conducted a large epidemiologic study on the prevalence and effect of *F. hepatica* infection among children from 26 communities in the Cusco region of Peru; ≈10% of children had evidence of *Fasciola* infection (*16*). Children given a diagnosis of fascioliasis in that study were provided open-label treatment with triclabendazole. We retrospectively describe the outcomes of triclabendazole treatment among children with chronic fascioliasis from rural communities in Cusco, Peru.

## Materials and Methods

### Study Population

The initial study cohort consisted of children with chronic fascioliasis from 26 communities of the Ancahuasi, Zurite, and Anta Districts of the Cusco region in Peru (*16*). Informed consent was provided by parents of 2,958 children 3–16 years of age who had no history of previous treatment for *F. hepatica* infection to participate in an epidemiologic study. We studied children for evidence of *F. hepatica* infection by using a serum Fas2 ELISA for *Fasciola* antibodies (Bionoma, https://www.dnb.com) and microscopy of 3 consecutive stool samples by using 1 Kato-Katz test and 1 Lumbreras sedimentation test per specimen. The Lumbreras sedimentation test has been demonstrated to have high sensitivity for detecting helminths ova in human feces (*17*). We evaluated the likelihood of living under the US $3.75/day poverty line by using the Simple Poverty Scorecard validated for Peru (*18*). We calculated this likelihood by comparing the score obtained in a standardized household questionnaire against a table of probabilities of living under a certain poverty line assigned to questionnaire score intervals (*18*).

### Intervention

The Ministry of Health offered triclabendazole treatment to all children who had >1 positive test result for *F. hepatica* infection. We defined chronic fascioliasis as having *Fasciola* eggs in >1 stool sample. Children who had chronic fascioliasis and received directly observed treatment with >1 triclabendazole dose and attended follow-up were included in the retrospective cohort. We defined a triclabendazole dose as the oral administration of 10 mg/kg with a fatty meal (≈350 calories).

The local Ministry of Health provided 250 mg scored triclabendazole tablets (Egaten; Novartis Pharma AG, https://www.novartis.com). The number of triclabendazole doses was determined by an expert panel advising the Ministry of Health with no input from the investigators. The selection was based on age, weight, and the number of treatment rounds previously received. Doses were rounded up to the next half or full tablet. Children whose parents did not consent for treatment before any round were referred to the local health center for follow up.

### Assessment of Response

We tested children who received >1 dose of triclabendazole for treatment response by using microscopy for 3 stool samples collected between 1 and 4 months after treatment. We assessed response to treatment by using parasitologic cure and egg reduction rate. We defined parasitologic cure as the absence of *Fasciola* eggs in 3 stool samples each tested by using 1 Kato Katz test and 1 Lumbreras rapid sedimentation test (*17*). The arithmetic mean for the Kato Katz egg count was calculated for each child by using the values from the 3 stool samples tested. Some children who had negative Kato Katz test results (0 eggs/gram of stool) were given a diagnosis of chronic fascioliasis on the basis of only the Lumbreras rapid sedimentation test. We calculated the geometric mean egg count for the population before and after each treatment. The egg reduction rate (ERR) was calculated by using the formula: ERR = (geometric mean pretreatment egg count – geometric mean posttreatment egg count)/(geometric mean pretreatment egg count) × 100 and was presented as a percentage. Children who did not achieve parasitologic cure were offered additional treatment courses as needed to achieve cure. After the fourth triclabendazole treatment, children were considered to have failed treatment with triclabendazole and to harbor drug-resistant *F. hepatica* parasites.

### Statistical Analysis

We used SPSS Statistics 25.0 (IBM Corp., https://www.ibm.com) for statistical analysis. For univariate analysis, we calculated frequencies, mean ± SD, median with interquartile range (IQR), and geometric means with 95% CIs to determine the distribution of the variables. Parasitologic cure after each round of treatment was recorded and used to estimate the efficacy of triclabendazole. We defined efficacy as the proportion of all children who were cured after each round and treatment regimen and calculated the egg reduction rate for each treatment round. Children who were lost to follow-up or whose parents refused further testing after treatment were excluded from the analysis of efficacy. We compared demographic information, socioeconomic status, and epidemiologic information between children with parasitologic cure and children without cure by using the Student t-test, Mann-Whitney U test, or χ^2^ test when appropriate. A p value <0.05 was considered significant for all statistical tests.

### Ethics

The study was approved by the Institutional Ethics Committee of Universidad Peruana Cayetano Heredia and the University of Texas Medical Branch. Informed consent was obtained in Quechua or Spanish language from the children’s parents or guardians. In addition, children >6 years of age provided verbal assent before any study procedure in the parent study.

## Results

A total of 228 (7.7%) of 2,958 children had >1 positive test result for *Fasciola*. A total of 166 (5.6%) children were passing eggs in the stool and met criteria for chronic fascioliasis, and 146 (88%) children met criteria to participate in the retrospective cohort (Figure). Most (77/146, 53.0%) children female; mean ± SD age was 10.4 ± 3.1 years (Table 1) The geometric mean egg count was 25 (95% CI 19.5–32.2) eggs/gram of stool (range 0‒820 eggs/gram of stool). Twenty children had an egg count of 0 eggs/gram of stool by the Kato Katz test but were given a diagnosis by the Lumbreras rapid sedimentation test, which is not quantitative. The median eosinophil count was 290 cells/μL (IQR 195–425 cells/μL). A total of 72% (104/146) of the children had a positive Fas2 ELISA result for *Fasciola* antibodies.

**Figure:**
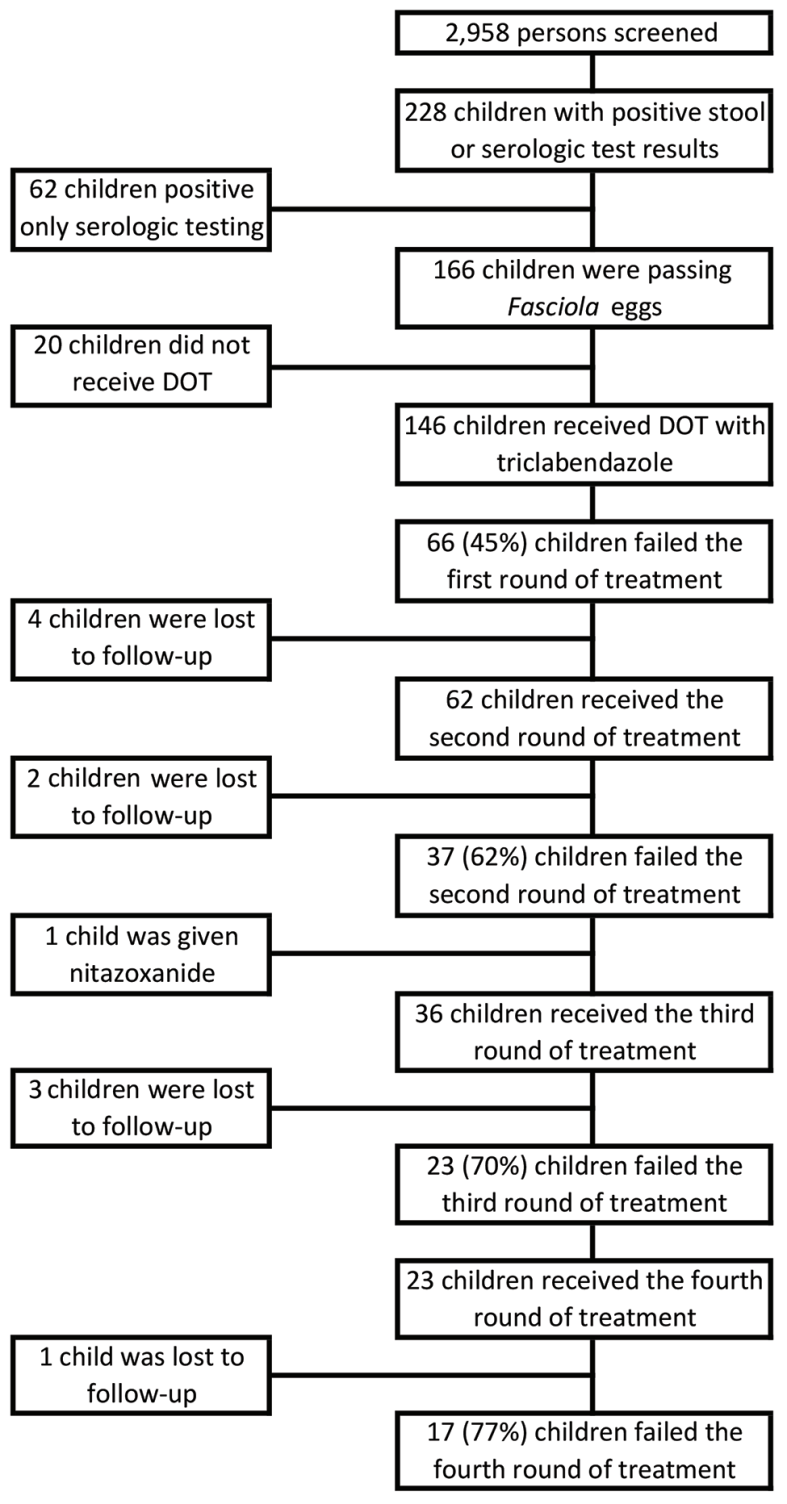
Flowchart of selection and treatment for participants in study of triclabendazole treatment failure for *Fasciola hepatica* infection among preschool and school-age children, Cusco, Peru. DOT, directly observed therapy.

**Table 1:** Characteristics of participants in study of triclabendazole treatment failure for *Fasciola hepatica* infection for 146 preschool and school-age children, Cusco, Peru*

Characteristic	Value
Sex	
F	77 (52.7)
M	69 (47.3)
District	
Ancahuasi	81 (55.5)
Anta	53 (36.3)
Zurite	12 (8.2)
Other parasites†	
0	83 (56.8)
1	47 (32.2)
2	16 (11.0)
Fas2 ELISA test result‡	
Positive	104 (72.7)
Negative	39 (27.3)
Likelihood of poverty‡§	
<50%	112 (83.0)
>50%	23 (17.0)
Eosinophil count, cells/µL‡	290 (195–425)
Baseline hemoglobin, g/dL¶	12.9 (12.2–13.7)
Baseline (IQR) HAZ‡	−1.57 (−2.16 to −0.94)
Median age ± SD, y	10.4 (± 3.14)
Geometric mean (95% CI) baseline egg count/g of stool	25 (19.5–32.2)

*Values are no. (%), median (IQR), or mean ± SD unless otherwise indicated. HAZ, height-for-age Z score; IQR, interquartile range. †Gastrointestinal parasites. ‡Does not add up to 146 because of missing data. §Likelihood of living at a level <US $3.75/day. ¶Uncorrected at enrollment.

### First Treatment

During the initial round of treatment, 139/146 (95.2%) children received 1 dose of triclabendazole and 7/146 (4.8%) children received 2 doses separated by 24 hours. The median number of days from treatment to assessment of response was 88 (IQR 56–114). Overall, 80/146 (55%) children achieved parasitologic cure after the first round of treatment, including 75/139 (54%) treated with 1 dose of triclabendazole and 5/7 (71%) treated with 2 doses (Table 2).

**Table 2:** Triclabendazole treatment outcomes per round for study of triclabendazole treatment failure for *Fasciola hepatica* infection for 146 preschool and school-age children, Cusco, Peru

Round	Regimen, no. doses	Parasitologic cure, no. (%)	Parasitologic failure, no. (%)
1	1	75 (54)	64 (46)
	2	5 (71)	2 (29)
	Overall	80/146 (55)	66/146 (45)
2	1	5 (46)	6 (55)
	2	17 (37)	29 (63)
	>2	1 (33)	2 (67)
	Overall	23/60 (38)	37/60 (62)
3	1	0 (0)	3 (100)
	2	2 (67)	1 (33)
	>2	8 (30)	19 (70)
	Overall	10/33 (30)	23/33 (70)
4	2	0 (0)	3 (100)
	>2	5 (26)	14 (74)
	Overall	5/22 (22.7)	17/22 (77.3)

Children who had a positive pretreatment Fas2 ELISA result (p = 0.026) and a higher egg count (p = 0.001) were more likely to fail the first round of triclabendazole (Table 3). The baseline geometric mean egg count of children who achieved parasitologic cure was less than half the geometric mean egg count of children who failed to be cured (17, 95% CI 11.9‒24.5 vs. 40.9, 95% CI 30.3–55.4) eggs/gram of stool. No significant differences by age (p = 0.76), sex (p = 0.54), district (p = 0.9), socioeconomic score (p = 0.54), baseline height-for-age Z score (p = 0.19), or baseline hemoglobin level (p = 0.97) were evident between those who failed treatment and those who responded to the first round of treatment. The overall ERR after the first round of treatment was 84.8%. The ERR among those who failed the first round of treatment was 53%.

**Table 3:** Response to first round of triclabendazole treatment for *Fasciola hepatica* infection among preschool and school-age children compared with failure to respond to first or fourth round of treatment, Cusco, Peru*

Characteristic	Cured after first round, n = 80	Failed after first round, n = 66	p value†	Failed after fourth round, n = 17	p value†
District					
Anta	27 (34)	26 (39)	0.48	12 (71)	0.005
Other	53 (64)	40 (61)		5 (29)	
Fas2 ELISA result					
Negative	27 (35)	12 (18)	0.02	2 (12)	0.08
Positive	50 (65)	54 (82)		15 (88)	
Likelihood of poverty‡					
<50%	56 (70)	56 (89)	0.08	17 (100)	0.03
>50%	16 (30)	7 (11)		0 (0)	
Other parasites§					
No	42 (53)	41 (62)	0.24	11 (65)	0.35
Yes	38 (48)	25 (38)		6 (35)	
Sex					
F	44 (55)	33 (50)	0.54	10 (59)	0.77
M	36 (45)	33 (50)		7 (41)	
Age, y, mean ± SD	10.5 ± 2.9	10.4 ± 3.4	0.85	9.0 ± 3.6	0.06
Baseline egg count, eggs/g of stool	33.3 (6.6–53.3)	48.3 (25–87.5)	0.001	60 (26–100)	0.005
Baseline hemoglobin, g/dL¶	12.9 (12.1–13.7)	12.9 (12.3–13.4)	0.97	12.3 (12–13)	0.08
Baseline HAZ#	−1.6 (−2.2 to −1.0)#	−1.5 (−2.0 to −0.9)#	0.19	−1.2 (−1.9 to −0.7)	0.17
Eosinophil count, cells/μL	300 (190–460)#	265 (197–412)	0.36	310 (230–500)	0.64

*Values are no. (%) or median (interquartile range) except as indicated. HAZ, height-for-age Z score. †By Mann-Whitney U test. ‡Likelihood of living under US $3.75/day poverty line (*18*). §Gastrointestinal parasites. ¶Uncorrected at enrollment. #One or 2 persons were missing information for this variable.

### Second Treatment

A total of 4 (6.1%) of the 66 children eligible to receive a second round of treatment were lost to follow-up or their parents refused further treatment. The geometric mean egg count before the second round of treatment was 19 (95% CI 12.3–29.5) eggs/gram of stool (range 0‒287 eggs/gram of stool). During the second round, 11 (17.7%) of 62 children received 1 dose of triclabendazole, 48 (77.0%) of 62 received 2 doses, and 3 (4.8%) of 62 received >2 doses. The median time between the second round of treatment and the assessment of response was 49 (IQR 34–65) days. Overall, 23 (38.0%) of 60 children achieved parasitologic cure after the second round of treatment, including 5 (46.0%) of 11 children who received 1 dose, 17 (37.0%) of 46 children who received 2 doses, and 1 (33.0%) of 3 children who received >2 doses. Two children did not provide stool samples for assessment of parasitologic cure. The overall ERR after the second round of treatment was 60.6%. However, the ERR for those who failed the second round of treatment was only 4.6%.

### Third Treatment

A total of 36 (97.3%) of the 37 children eligible for a third round of treatment received triclabendazole again and 33 (83.3%) of 36 children were prescribed treatment regimens containing >2 doses (Table 2). The geometric mean egg count before the third round of treatment was 35.8 (95% CI 22.9–56.4) eggs/gram of stool (range 0‒387 eggs/gram of stool). Three children did not provide follow-up specimens. Parasitologic cure was achieved by 10 (30%) of 33 children. One child did not receive triclabendazole and was given nitazoxanide (500 mg 2×/d for 6 d) without achieving parasitologic cure. The overall ERR after the third round of treatment was 58%, and the ERR of those who failed the third round was 34.7%.

### Fourth Treatment

A total of 23 children who failed treatment again were administered >2 doses of triclabendazole for a fourth round of treatment. One child did not provide a follow-up specimen. The pretreatment geometric mean egg count was 29.6 (95% CI 16–55.3) eggs/gram of stool (range 0‒833 eggs/gram of stool). Parasitologic cure was achieved by 5 (23.0%) of 22 children. The overall ERR after the fourth round of triclabendazole was 23.6%. For children who were not cured, the geometric mean egg count increased by 89.3%.

### Triclabendazole Failure

A total of 17 (11.6%) of 146 children given triclabendazole were considered to have failed triclabendazole treatment and harbored drug-resistant *F. hepatica* parasites. The median age of the children was 8.5 (IQR 6.4– 12.3) years and 10 (58.8%) of 17 were female. All households had less than a 50% likelihood of living under a US $3.75/day poverty line. Almost one third (6/17, 35.3%) were infected with other gastrointestinal parasites at enrollment. A total of 11 (65.0%) of 17 children received additional treatment courses without achieving parasitologic cure (Table 4). Children who were cured after the first round of treatment when compared with children who had drug-resistant parasites were less likely to live in Anta district, more likely to live under the poverty line, and more likely to have a lower baseline egg count (Table 3).

**Table 4:** Clinical characteristics of 17 children who failed 4 rounds of triclabendazole treatment for *Fasciola hepatica* infection among preschool and school-age children, Cusco, Peru*

Child	Community	District	Age, y/sex	Other parasites	HAZ	Living under US $3.75/day	ERR, %†	Total drug dose, mg/kg‡	Additional treatment received
1	Anta	Anta	6.8/F	Yes	−0.6	25	0§	90	1 more round
2	Anta	Anta	14.1/M	No	−2.3	25	40	90	2 more rounds
3	Conchacalla	Anta	10.5/M	No	−1.8	13	67	210	Nitazoxanide
4	Conchacalla	Anta	3.2/F	No	−2.4	36	40	50	2 more rounds
5	Conchacalla	Anta	6.0/F	No	−0.4	36	0§	210	None
6	Inquilpata	Anta	8.5/M	No	−0.9	36	30	50	2 more rounds
7	Inquilpata	Anta	9.7/F	No	−1.2	36	43	50	2 more rounds
8	Inquilpata	Anta	3.3/M	Yes	−1.2	49	+275	210	None
9	Izcuchaca	Anta	13.9/F	No	−0.6	36	+574	150	1 more round
10	Izcuchaca	Anta	7.4/M	No	−0.6	25	63	150	1 more round
11	Mantoclla	Anta	7.3/F	Yes	−2.0	25	10	150	None
12	Mantoclla	Anta	9.2/F	Yes	−1.9	25	31	150	None
13	Caccahuara	Ancahuasi	13.1/F	No	−1.5	25	17	210	Nitazoxanide
14	Caccahuara	Ancahuasi	15.5/F	No	−1.9	13	+50	210	None
15	Caccahuara	Ancahuasi	7.8/M	No	−1.1	25	+100	210	Nitazoxanide
16	Chaquillccasa	Ancahuasi	11.5/M	Yes	−0.9	25	+250	210	None
17	Chaquillccasa	Ancahuasi	5.5/F	Yes	−2.1	49	63	210	1 more round

*ERR, egg reduction rate; HAZ, height-for-age Z score. †Between baseline and after the fourth round of treatment, as estimated by the number of eggs in Kato Katz tests. + signs indicate the percent increase in the egg count. ‡Total cumulative dose received after the fourth round of treatment §Diagnosed only by Lumbreras rapid sedimentation test and a negative Kato Katz test result.

## Discussion

Fascioliasis imposes a large burden on impoverished human populations, and triclabendazole is the only medication recommended for treatment and control. In our cohort of children who had chronic fascioliasis, the efficacy of triclabendazole was low, especially after treatment with a single dose. The overall drug efficacy after 1 round of treatment was 55%, and it decreased further after subsequent rounds to 38%, 30%, and 23%, respectively. Overall, 11% of the children failed to respond to >4 triclabendazole treatment rounds. The overall ERR rates were lower after each round of treatment and modest in children who failed each triclabendazole round. Higher pretreatment egg counts, higher socioeconomic status, and living in the Anta district were associated with failure to achieve parasitologic cure.

Factors underlying failure of triclabendazole treatment for chronic fascioliasis might include poor medication quality and bioavailability. We used Egaten in this study, which is the human formulation of triclabendazole donated by Novartis Pharma to the Peruvian Ministry of Health. This medication was administered to children well before its expiration date. In addition, children received a standard fatty meal before each triclabendazole dose. Food increased triclabendazole bioavailability between 2-fold and 3-fold in pharmacokinetic studies (*8*). However, scarce information on triclabendazole population pharmacokinetics in *Fasciola*-infected persons from disease-endemic areas is available.

We based the chronic *Fasciola* diagnosis on the presence of eggs in stool and tested for parasitologic cure at 1–4 months posttreatment by using the same type and number of stool tests as those used to diagnose the infection. This period is within the duration of the migratory phase of *Fasciola* parasites when immature parasites have not reached the bile ducts or produce eggs. Thus, our approach in an area with moderate prevalence of fascioliasis and low intensity of infection decreased the possibility of reinfection as a cause of persistent positive results for stool microscopy (*19*).

The poor response to triclabendazole contrasts with previous reports from the highlands of South America. Maco et al. reported parasitologic cure rates of 100% after 2 doses of 7.5 mg/kg of triclabendazole in 24 hours and 95% after a single dose of 10 mg/kg of triclabendazole among children with fascioliasis in highlands of Peru (*5*). A study in the Altiplano of Bolivia, near the border with Peru, reported parasitologic cure rates of 78% and 98% among chronically infected children after 1 and 2 rounds of treatment with a single 10 mg/kg dose of triclabendazole, respectively (*3*). No recent large treatment studies are available from South America with which to compare our results, but increasing reports of triclabendazole failure in the area support the idea of decreasing effectiveness and raise concern for the lack of alternative medications.

A higher pretreatment egg count was found for children who failed >1 rounds of treatment. This observation has been described for other trematode infections, such as schistosomiasis. Black et al. studied a cohort of 200 men who had occupational exposure to schistosomiasis in Lake Victoria, Kenya, and proposed that even with drug efficacies >90%, persons who had a high burden of infection might still be infected with enough surviving egg-producing parasites to have positive results for stool microscopy (*20*). The mesoendemic area we studied showed a moderate *Fasciola* prevalence, and egg burden was not as high as those described in hyperendemic areas around Lake Titicaca, where the egg count geometric mean can reach 700 eggs/gram of stool (*21*). For that reason, it is unlikely that triclabendazole failure could be explained by surviving parasites despite high drug efficacy.

Survival of juvenile parasites migrating through the liver has been proposed as a cause for persistent infection after treatment. In *Fasciola* infection models, juvenile parasites have reduced susceptibility to triclabendazole compared with established infection with mature parasites (*22*). No clinical trials of triclabendazole treatment of acute fascioliasis have been published, and the clinical experience in published case series is inconsistent. After 2 triclabendazole doses, some authors reported low efficacy (Ramadan et al., 55% efficacy [*23*]) and some authors reported high efficacy (Chen et al., 96% efficacy [*24*]). However, ascertainment bias is a major issue in acute fascioliasis case series because there is no consensus on case definition and treatment response measures. In our community-based study, only 0.4% of children had acute fascioliasis defined by negative stool test results, positive results for *Fasciola* antibodies, eosinophilia (>500 cells/μL), and elevated levels of aminotransferases (*16*). Thus, we believe that the potential contribution of the survival of migrating parasites to the persistence of infection in our treatment cohort is negligible.

Persons from Anta district and with <50% chance of living in poverty were more likely to fail a fourth round of treatment. The Anta district is the district closest to Cusco and has one of the lowest poverty levels in the province (*25*). We hypothesize that access to veterinary triclabendazole to give to cattle might be associated with higher urbanization and socioeconomic status in our cohort. It is likely that drug resistance emerges first in livestock under constant triclabendazole selective pressure but also under inconsistent dosing because of lack of quality control of veterinary products or training of farmers (*14**,**26**,**27*). Subsequently, the proportion of drug-resistant parasites shed by cattle in the environment might reach a threshold, leading to transmission to humans. However, further studies are needed to evaluate the association between drug resistance in cattle, environmental contamination with drug-resistant isolates, clonal expansion in intermediate hosts, and the emergence of triclabendazole resistance in humans.

A major limitation of this study was the lack of established dosing schemes. A consultant from the Ministry of Health made dosing decisions after reviewing the child’s age, weight, and previous treatment courses. These decisions were outside the control of the study investigators and introduced some heterogeneity in the dosing. However, after failing triclabendazole, some children received treatment with multiple doses, well above the recommended dosing regimens in the triclabendazole package insert. We did not note increased effectiveness with regimens containing >2 doses of triclabendazole, but the small numbers treated precluded a statistical comparison. A more cautious approach was used with children <6 years of age because fewer data support the safety of triclabendazole for this age group.

A potential limitation when evaluating drug ef-ficacy is in the ability to distinguish persistent infec-tions from reinfections in disease-endemic areas. In addition, the low sensitivity of stool microscopy and variability of egg shedding could hinder ascertainment of treatment outcomes, particularly ERR. Testing >1 stool sample and combination of microscopy methods was necessary to increase sensitivity. The timing for assessment of cure for fascioliasis has not been well established. Intervals between determination of treatment failure and the next round of treatment lasted several months for some children because of triclabendazole shortages. Thus, we cannot exclude increased parasite burdens before treatment among children caused by rapidly occurring reinfections. However, considering that we studied an area with a moderate infection prevalence, these reinfections probably occurred rarely if at all.

There are no established alternatives to triclabendazole for treating of fascioliasis. Nitazoxanide has been used in some studies. This drug was used for children who had repeated triclabendazole treatment failures but was not effective. In Egypt, Ramadan et al. reported a cure rate of 30% for nitazoxanide among persons who had acute fascioliasis who failed 2 triclabendazole doses (*23*). There are wide regional variations in reported nitazoxanide cure rates among *Fasciola*-infected persons (*28**,**29*). We previously reported a case series of triclabendazole-resistant fascioliasis in Cusco and noted a lack of nitazoxanide effectiveness among persons previously failing multiple triclabendazole treatments (*14*). Although we provided treatment with nitazoxanide to only 4 children with nitazoxanide in the current cohort study, our results do not support the use of nitazoxanide as rescue treatment for persistent *Fasciola* infections.

In this cohort, we have demonstrated decreased response rates to triclabendazole among children with chronic fascioliasis, including poor egg reduction rates and low parasitologic cure rates with single triclabendazole doses. In addition, we have documented high levels of drug resistance in children treated several times with increasing doses of triclabendazole. The absence of effective alternative medications and lack of interventions to overcome drug-resistance mechanisms are of concern in disease-endemic areas. Triclabendazole use stewardship in humans and livestock is urgently needed to prevent further decrease in infectiveness. In this setting, it is unclear whether mass drug administration control strategies might aggravate the problem. Ongoing research in the Cusco area is evaluating the mechanisms of triclabendazole resistance. Clinicians should be aware of alternative drugs and the interactions between triclabendazole-resistant parasites in livestock, human, and the environment that drive transmission to children.
